# Human Umbilical Cord MSC Delivered-Soluble TRAIL Inhibits the Proliferation and Promotes Apoptosis of B-ALL Cell In Vitro and In Vivo

**DOI:** 10.3390/ph15111391

**Published:** 2022-11-11

**Authors:** Fangshan Chen, Xianmei Zhong, Qian Dai, Kuo Li, Wei Zhang, Jie Wang, Yueshui Zhao, Jing Shen, Zhangang Xiao, Hongyun Xing, Jing Li

**Affiliations:** 1 Department of Oncology and Hematology, The Affiliated Traditional Chinese Medicine Hospital of Southwest Medical University, Luzhou 646000, China; 2 Laboratory of Molecular Pharmacology, Department of Pharmacology, School of Pharmacy, Southwest Medical University, Luzhou 646000, China; 3 Cell Therapy & Cell Drugs of Luzhou Key Laboratory, Luzhou 646000, China; 4 South Sichuan Institute of Translational Medicine, Luzhou 646000, China; 5 Department of Pharmacy, People’s Hospital of Nanbu County, Nanchong 637300, China; 6 Department of Hematology, The Affiliated Hospital of Southwest Medical University, Luzhou 646000, China

**Keywords:** mesenchymal stem cells, B-cell acute lymphocytic leukemia, TRAIL, proliferation, apoptosis

## Abstract

The TNF-related apoptosis-inducing ligand (TRAIL) could induce apoptosis of leukemic cells, while showed no cytotoxic effect on normal cells. One of the limitations for application of recombinant TRAIL (rhTRAIL) in leukemia treatment is that the serum half-life of this protein is short. Gene delivery is a good strategy to prolong the half-life of TRAIL. In this study, we genetically engineered umbilical cord-MSCs to continuously express and secrete soluble TRAIL (MSC-sTRAIL), to investigate the effects of MSC-sTRAIL on B-cell acute lymphocytic leukemia (B-ALL) cells. In vitro, MSC-sTRAIL significantly inhibited the proliferation of B-ALL cells by suppressing PI3K/AKT and MEK/ERK signaling pathways, and induced apoptosis of B-ALL cells via the caspase cascade-mediated pathway and mitochondrial-mediated pathway. In vivo, MSC-sTRAIL dramatically inhibited B-ALL cell growth. Meanwhile, B-ALL-induced splenic and renal injuries were significantly alleviated after MSC-sTRAIL treatment. Moreover, the serum levels of MSC-secreted sTRAIL were still high in MSC-sTRAIL treated mice, indicating an extended half-life of sTRAIL. Our study suggests that MSC delivered-TRAIL secretion is a potential therapeutic strategy for B-ALL treatment.

## 1. Introduction

Acute lymphoblastic leukemia (ALL) is the most common hematologic malignancy, and is one of the leading cause of cancer-related deaths in the pediatric population [1]. B-cell ALL (B-ALL) and T-cell ALL (T-ALL) account for approximately 85% and 15% of all pediatric ALL cases, respectively [2]. The most common and economical therapy option for B-ALL is chemotherapy [3]. In recent years, with the development of gene editing and deep-sequencing technologies, cell immunotherapy has become a promising option for B-ALL treatment [4]. For example, more and more clinical trials have shown that CD19-directed chimeric antigen receptors (CARs)-T cells treatment can achieve 70–90% complete remission in the treatment of pediatric B-ALL [5]. The major limitation of cell immunotherapy is the adverse effects, such as cytokine response storm (CRS), which limits the clinical benefit [6].

The TNF-related apoptosis-inducing ligand (TRAIL), a member of the TNF superfamily, can inhibit tumor cell growth by initiating apoptotic pathways in many types of solid and hematologic cancers, while without affecting normal cells [7,8]. The activation of PI3K/AKT and MEK/ERK signaling pathways are important for the proliferation of cancer cells [9,10], and studies have shown that TRAIL-induced cancer cell death is mainly mediated by the suppression of PI3K/AKT signaling pathway [11,12], and by initiating apoptotic pathway [13,14]. The apoptosis process of cells is mainly mediated by two pathways, the extrinsic receptor-mediated pathway and intrinsic mitochondria-mediated pathway [15]. Among these two apoptotic pathways, the extrinsic pathway is mainly mediated by binding of the ligand with a specific receptor, such as the binding of TRAIL with TRAIL receptor, which could induce the downstream activation of apoptotic proteins caspase 8, caspase 9, and caspase 3 [15]; the intrinsic pathway is mitochondrial-mediated pathway, and is mainly mediated by the insertion of Bax protein into the mitochondrial membrane to induce the release of cytochrome c; cytochrome c binds with caspase 9, and in turn induces the activation of caspase-3 cascade and apoptosis of the cell [15]. Previous study has shown that TRAIL could induce apoptosis of cancer cells by regulating both apoptotic pathways [16,17].

TRAIL also can be used in combination with other therapeutic methods, such as chemotherapeutic agents and radiation, to synergically induce the maximum apoptotic rate in cancer cells [18]. The recombinant human TRAIL (rhTRAIL) was clinically used for cancer treatment but later the application became limited, as the serum half-life of this protein is short, and the efficacy of this drug is not satisfying [19]. Since the short half-life of TRAIL caused a need to continuously use this drug to achieve the effective concentrations, new strategies to prolong the half-life of TRAIL need to be investigated [20]. Gene delivery might be a good strategy. Using the adeno-associated virus- or lentivirus-mediated overexpression method to genetically modify B-cell non-Hodgkin lymphoma (B-NHL) cells to produce secretable TRAIL (sTRAIL), Yuan et al. found that TRAIL gene expression driven by CD20 promoter could inhibit the proliferation of B-NHL cells in vivo and in vitro [21]. Clodi et al. found that precursor B-ALL cells showed modestly sensitivity to TRAIL, which was not associated with the expression pattern of TRAIL receptors [22].

One of the attractive vehicles for gene delivery is MSCs [23]. MSCs are suitable vectors for cancer gene therapy, as they showed many properties, including easy accessibility, low immunogenicity, homing to target site, and high capacity for gene transduction [24]. TRAIL-transduced MSCs have been used as an anticancer method in solid cancers [25]. For hematological cancers, Yan et al. generated scFvCD20-sTRAIL fusion gene expressed-umbilical cord-derived MSCs to kill NHL lymphoma cells, and found that the scFvCD20-sTRAIL secreting MSCs could inhibit the proliferation of tumor cells in vitro, and inhibit tumor development in vivo [26]. Hence, this strategy is a potential therapeutic method for hematologic cancer treatment.

Whether TRAIL regulates B-ALL cell activity is still unclear. In the present study, we used rhTRAIL, and designed a TRAIL gene delivery system, in which human umbilical cord derived-MSCs (UC-MSCs) were engineered to generate sTRAIL. The effectiveness of this system against B-ALL cells were evaluated in vitro and in vivo. We found that both rhTRAIL and UC-MSC secreted TRAIL could inhibit B-ALL cells proliferation in vitro and in vivo, while UC-MSCs secreted TRAIL exhibited longer serum half-life in vivo.

## 2. Results

### 2.1. rhTRAIL Inhibits the Proliferation of B-ALL Cells

To assess the effect of rhTRAIL on B-ALL cells, we treated human B-ALL cells, Nalm-6 and Sup-b15 cells, with different doses of rhTRAIL, and checked the cell activities by CCK-8 assay. We found that a high concentration of rhTRAIL or longer time of treatment induced stronger suppression effects on cell proliferation of both Nalm-6 and Sup-b15 cell (Figure S1).

### 2.2. Gene Modified UC-MSCs Can Secrete Soluble TRAIL (sTRAIL) without Apparent Interference on Their Biological Properties

**Figure 1:**
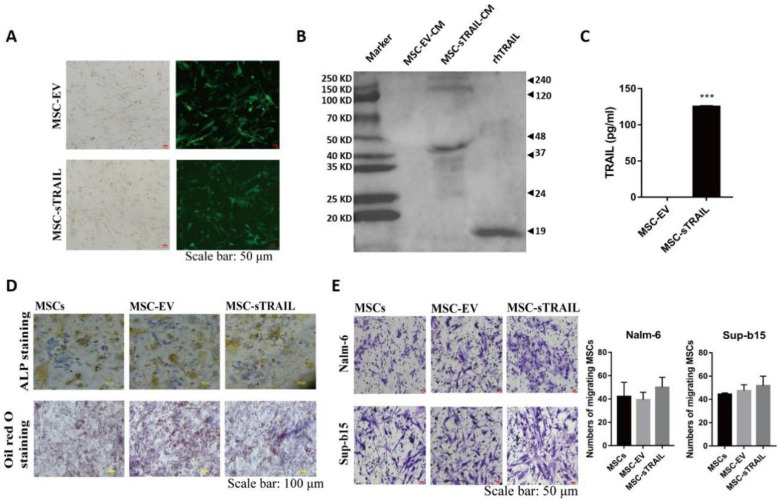
The successful construction of MSC-sTRAIL and lentiviral infection do not affect the biological properties of MSCs. After infecting MSCs with lentivirus: (**A**) White light and fluorescence micrographs of MSCs at day 5 after lentiviral infection (Scale bar 50 μm). (**B**) The expression of TRAIL protein in MSC-sTRAIL-CM on the fifth day of infection was measured by western blotting. (**C**) The concentration of TRAIL in conditional medium was determined by ELISA (n = 3 per group; Student’s *t* test, ***, *p* < 0.001, MSC-sTRAIL-CM group compared with MSC-EV-CM group). (**D**,**E**) Staining plots for induction of adipogenic and osteogenic differentiation and homing of MSCs, MSC-EV, and MSC-sTRAIL (Scale bar: 100 μm, and 50 μm for (**D**,**E**), respectively).

The lentiviral sTRAIL expression vector was successfully generated (Supplementary Figure S2). To verify whether this vector could express soluble TRAIL, UC-MSCs were transduced with control or sTRAIL-overexpression viruses to obtain MSC-EV (control) and MSC-sTRAIL (TRAIL overexpression) cells, respectively. As shown in Figure 1A, both groups could express GFP protein.

Next, we checked the time kinetics of TRAIL secretion; the MSC-EV and MSC-sTRAIL cells were placed on 100-mm cell culture dishes, then we collected conditioned medium after 24 h, 48 h, 72 h, 96 h, and 120 h of plating, respectively. Then, we checked the levels of TRAIL by western blotting, as shown in Supplementary Figure S3. The secretion levels of sTRAIL of MSC-sTRAIL cells were increased in a time-dependent manner, with highest levels after 120 h of cell plating. Meanwhile, we found multiple molecular weight (MW) bands at MW site of 24, 48, 72, 120, and 240 kDa in MSC-sTRAIL group, which were similar results to those reported by other researchers [27], while MSC-EV group showed no band (Supplementary Figure S3 and Figure 1B), indicating that MSC-sTRAIL cell could secrete multimers of soluble TRAIL. Further assay showed that 120 h after the cells were plated on cell culture plates, the TRAIL level of MSC-sTRAIL group was 125.9 pg/mL, while TRAIL was undetectable in MSC-EV group (Figure 1C).

To investigate whether the biological properties of MSCs was affected after lentivirus infection, we examined the ability of MSC osteogenesis and adipogenesis by ALP staining and Oil Red O staining, respectively. There was no significant difference in the ability of adipogenic and osteogenic differentiation between the MSCs group, MSC-EV group, and MSC-sTRAIL group (Figure 1D). Next, we investigated the homing property of MSC, We found that sTRAIL secreted-MSC could migrate towards B-ALL cells, Nalm-6, and Sup-b15, which were similar effects as those found in the MSCs group and MSC-EV group (Figure 1E). Overall, these data indicated that sTRAIL cannot change the biological properties of MSC.

### 2.3. MSC-sTRAIL Inhibits the Proliferation of B-ALL Cells In Vitro

**Figure 2:**
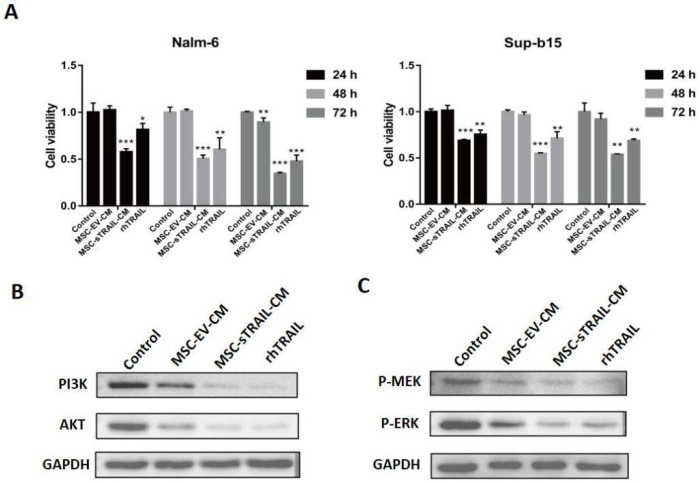
MSC-sTRAIL inhibits the proliferation of B-ALL cells. (**A**) MSC-sTRAIL inhibits the proliferation of Nalm-6 and Sup-b15 cells. CM: conditional medium (*n* = 6 per group; Student’s *t* test, *, *p* < 0.05, **, *p* < 0.01, ***, *p* < 0.001, MSC-EV-CM group, MSC-sTRAIL-CM group, and rhTRAIL compared with control group). Nalm-6 cells were treated without (control group) or with MSC-EV-CM, MSC-sTRAIL-CM, or rhTRAIL for 48 h, then, (**B**) protein levels of PI3K and AKT were investigated by western blotting, and GAPDH was used as the inner control. (**C**) Phosphorylation levels of MEK and ERK proteins were investigated by western blotting, and GAPDH was used as the inner control.

To determine the effect of MSC-sTRAIL on B-ALL cells, Nalm-6 and Sup-b15 cells were treated with MSC-sTRAIL conditional supernatant with TRAIL concentration of 125.9 pg/mL or rhTRAIL(100 ng/mL). As shown in Figure 2A, both of MSC-sTRAIL and rhTRAIL dramatically inhibit the proliferation of B-ALL cells, compared with the control groups. Meanwhile, sTRAIL secreted by MSC showed better inhibitory effects than rhTRAIL on B-ALL cell proliferation (Figure 2A). Studies have demonstrated that TRAIL can inhibit the proliferation of acute myeloid leukemia cells through the PI3K/AKT pathway [9]. We found that the protein levels of total PI3K, and AKT were all decreased (Figure 2B). As the activation of MEK/ERK signaling pathway is another key pathway for cancer cell proliferation [10], we next checked whether TRAIL regulates this pathway; we found that the phosphorylation levels of MEK (P-MEK), and ERK (P-ERK) proteins were all decreased after MSC-sTRAIL treatment, compared with the MSC-EV group, in Nalm-6 cells (Figure 2C). Overall, these data indicated that MSC-sTRAIL inhibits the proliferation of B-ALL cells by suppressing PI3K/AKT and MEK/ERK signaling pathways.

### 2.4. MSC-sTRAIL Induces Apoptosis of B-ALL Cells In Vitro

**Figure 3:**
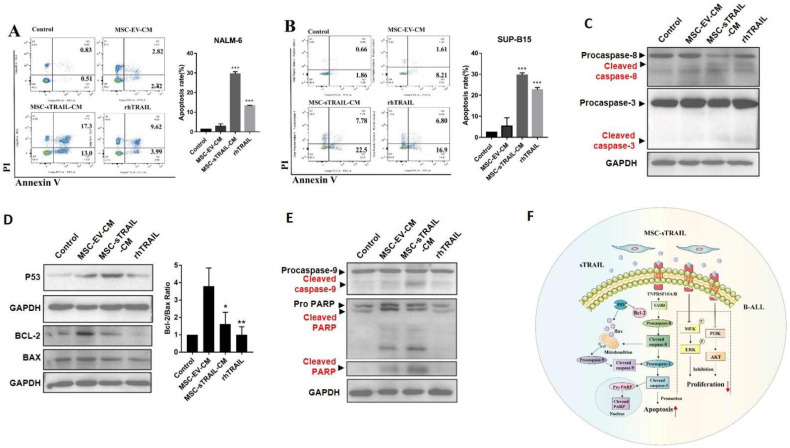
MSC-sTRAIL promotes apoptosis in B-ALL cell lines Nalm-6 and Sup-b15 cells. After treating Nalm-6 and Sup-b15 cells with MSC-EV-CM, MSC-sTRAIL-CM, or rhTRAIL for 72 h. (**A**,**B**) MSC-sTRAIL promotes the apoptosis of Nalm-6 and Sup-b15 cells. Nalm-6 cells were treated without (control group) or with MSC-EV-CM, MSC-sTRAIL-CM, or rhTRAIL for 48 h, then, (**C**) Protein levels of procaspase-8, cleaved caspase-8, procaspase3, and cleaved caspase-3 were investigated by western blotting, and GAPDH was used as inner control. (**D**) Protein levels of p53, Bcl-2, and Bax were investigated by western blotting, and GAPDH was used as inner control; the densitometry readings of each band were calculated by Image J software, then the ratio of Bcl-2/Bax were calculated and compared between each group. (**E**) Protein levels of procaspase-9, cleaved caspase-9, proPARP, and cleaved PARP were investigated by western blotting, and GAPDH was used as the inner control. (**F**) Summary diagram of the mechanism of how MSC-sTRAIL inhibits the proliferation and promotes apoptosis of B-ALL cells. (*n* = three independent experiments per group; Student’s *t* test, *, *p* < 0.05, **, *p* < 0.01, ***, *p* < 0.001, MSC-sTRAIL-CM group, and rhTRAIL group compared with MSC-EV-CM group).

Nalm-6 and Sup-b15 cells were treated with MSC-sTRAIL or rhTRAIL for 72 h and then apoptosis assay was performed. We found that, compared with the MSC-EV group, both MSC-sTRAIL-CM and rhTRAIL dramatically induced apoptosis of Nalm-6 cells (5.24% vs. 30.3% and 13.61%, respectively, *p* < 0.001) (Figure 3A) and of Sup-b15 cells (9.82% vs. 30.28% and 23.7%, respectively, *p* < 0.001) (Figure 3B).

Next, we investigated the mechanism of how MSC-sTRAIL or rhTRAIL induce the apoptosis of B-ALL cells. As TRAIL-induced cancer cell apoptosis could mediate by either caspase cascade pathway or mitochondria pathway [15], firstly we checked the change of cascade pathway, and we found that the levels of activation forms of apoptotic-related proteins, cleaved caspase 8, and cleaved caspase 3, were all increased in MSC-sTRAIL- or rhTRAIL- treated Nalm-6 cells, compared with control or MSC-EV group (Figure 3C). Next, we checked the protein levels of the mitochondria-related apoptotic pathway. Bcl-2 is an anti-apoptotic protein, and its expression could be suppressed by protein P53, and the decrease in Bcl-2/Bax ratio is a marker of mitochondria-mediated apoptosis [15]. We found that, after MSC-sTRAIL or rhTRAIL treated, the protein levels of P53 of Nalm-6 cells were increased, while the protein levels of Bcl-2 were decreased, accompanied by the decrease in Bcl-2/Bax ratio (*p* < 0.05, Figure 3D). Meanwhile, the activation form of caspase 9, cleaved caspase 9, and its downstream target- cleaved PARP protein- levels were increased in MSC-sTRAIL- or rhTRAIL-treated Nalm-6 cells, compared with control or MSC-EV group (Figure 3E). Overall, these results indicated that MSC-sTRAIL induces apoptosis of B-ALL cells in vitro by both extrinsic and intrinsic apoptosis pathways (Figure 3F).

### 2.5. MSC-sTRAIL Showed Little Effects on the Expression Levels of TRAIL Receptors of B-ALL Cells

It has been reported that rhTRAIL could downregulate the expression of TRAIL receptors [28], which in turn make tumor cells less sensitive to TRAIL [29], so, the maintenance of TRAIL receptor levels is key for TRAIL signal transduction. Next, we investigated whether MSC-sTRAIL regulates the expression of TRAIL receptors, TNFRSF10A, TNFRSF10B, and TNFRSF10D. We treated B-ALL cells, Nalm-6 and Sup-b15 cells, with rhTRAIL and MSC-sTRAIL for 24 h. We found that while rhTRAIL downregulated the mRNA levels of TRAIL receptors TNFRSF10A and TNFRSF10B, MSC-sTRAIL treatment had little effects on the expression levels of TRAIL receptors of B-ALL cells (Supplementary Figure S4).

### 2.6. MSC-sTRAIL Delivered in Mice Efficiently Control the B-ALL Cell Levels in Bone Marrow

**Figure 4:**
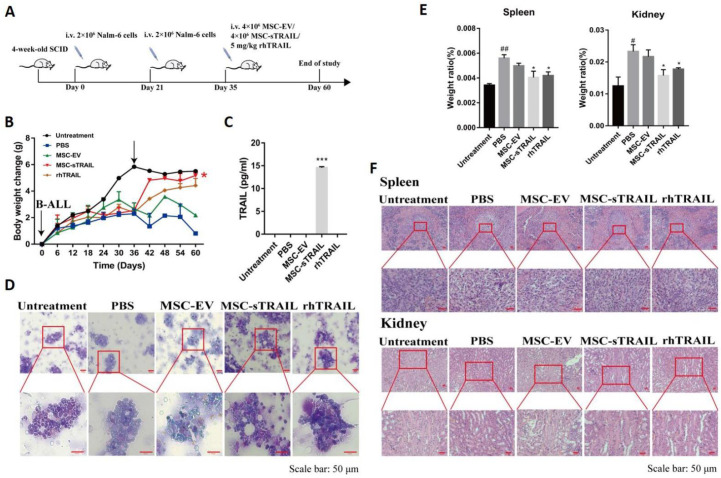
MSC-sTRAIL treats B-ALL-induced injury in mice. SCID mice were treated with 2 × 10^6^ Nalm-6 cells on day 0 and day 21, then on day 35, mice of untreated group, PBS group, MSC-EV group, MSC-sTRAIL group, and rhTRAIL group were each injected with 200 μL PBS, 200 μL PBS, 2 × 10^6^ MSC-EV cells, 2 × 10^6^ MSC, 5 mg/kg rhTRAIL, respectively. (**A**) in vivo experiment design. (**B**) MSC-sTRAIL inhibits B-ALL-induced weight loss in mice. (**C**) Serum TRAIL levels detected by ELISA. (**D**) Wright Giemsa staining of mouse bone marrow. (**E**) MSC-sTRAIL inhibits B-ALL-induced increase in specific gravity of spleen and kidney in mice. (**F**) H&E staining (Scale bar 50 μm. *n* = 5 per group; Student’s *t* test, *, *p* < 0.05, ***, *p* < 0.001, MSC-EV group and MSC-sTRAIL group compared with PBS group; #, *p* < 0.05, ##, *p* < 0.01, PBS group compared with untreated group. Untreated group: SCID mice not inoculated with Nalm-6 cells).

To investigate the therapeutic effect of MSC-sTRAIL on B-ALL in vivo, we established a B-ALL mice model using Nalm-6 cells. Figure 4A is scheme of the treatment design. On day 0 and day 21, the mice of PBS group, MSC-EV group, MSC-sTRAIL group, and rhTRAIL group were injected with Nalm-6 cells (2 × 10^6^/mouse). On day 35 after cell implantation, each mouse of the untreated group, PBS group, MSC-EV group, MSC-sTRAIL group, and rhTRAIL group were injected with 200 μL PBS, 200 μL PBS, 2 × 10^6^ MSC-EV cells, 2 × 10^6^ MSC, 5 mg/kg rhTRAIL, respectively, and kept for 25 days to collect tissues or serum for assay.

We found that the body weight of the mice inoculated with Nalm-6 cells decreased by about 5 g, compared with the uninoculated group, on day 35 after treatment (Figure 4B), and the mice appeared to be atrophied and less active, with arching of the back and reduced feeding (data not shown). The body weight gain of the mice of MSC-sTRAIL group were dramatically recovered to the levels of untreated mice only after 2 weeks of treatment (Figure 4B). rhTRAIL showed similar effects after 3 weeks of treatment, compared with the MSC-sTRAIL group (Figure 4B). After 25 days of treatment, the serum levels of TRAIL were undetectable in rhTRAIL-treated mice, while the levels of TRAIL in MSC-sTRAIL-treated group were still very high (14.46 pg/mL, Figure 4C), indicating a continuous expression of TRAIL protein in MSC-sTRAIL-treated mice.

To investigate the inhibitory effect of MSC-sTRAIL on B-ALL cell in vivo, 25 days after MSC-sTRAIL or rhTRAIL treatment, the bone marrow of mice of each group were stained with Richter-Gimza. Results revealed that a large number of Nalm-6 cells could be observed in the PBS group and MSC-EV group, but only a few Nalm6 cells were found in the bone marrow of mice of the MSC-sTRAIL-treated group and rhTRAIL group (Figure 4D). These results suggested that MSC secreted TRAIL could inhibit the proliferation of Nalm-6 cells in vivo.

### 2.7. MSC-sTRAIL-Delivery Alleviates B-ALL Cell-Induced Tissue Injuries in Mice

One of the major complications of acute leukemia is tissue injury, including acute kidney injury and splenomegaly [30,31]. We found that, after being inoculated with Nalm-6 cells, the weight of kidney and spleen were increased (*p* < 0.01, Figure 4E), while the weight of heart, liver, and lung had no change, compared with the control group (Supplementary Figure S5). Further H&E staining results showed that the white pulp structures of spleen of leukemic mice were loosely arranged and significantly damaged, and the texture of the medullary pay-off in the kidney was indistinct in PBS and MSC-EV group, whereas the spleen of MSC-sTRAIL and rhTRAIL-treated leukemic mice showed normal white pulp structure and clear renal medullary discharge texture (Figure 4F), indicating that MSC-sTRAIL and rhTRAIL play protective roles in the injury of kidney and spleen in B-ALL.

## 3. Discussion

Novel and effective therapeutic strategies are needed for B-ALL treatment. Here, for the first time, we demonstrate the possibility to reduce human B-ALL viability using gene modified human UC-MSC as a delivery vehicle to constantly express soluble TRAIL.

MSCs have been used as delivery vehicle for various anti-cancer drugs, such as interferon and interleukins, to improve the therapeutic efficacy [32,33]. In this study, we genetically modified UC-derived MSCs to secrete TRAIL protein by lentiviral transduction. Western blot results showed that, compared with the single TRAIL band found in rhTRAIL group, there are multiple bands for TRAIL protein in MSC-sTRAIL group with molecular weights of approximately 24, 48, 72, 120, and 240 kDa, which are the molecular weights of monomers to multimers of TRAIL, and are consistent with previous reported results [27]. Han et al. have reported that multibands are mixtures of posttranslational modification types and multimers of TRAIL protein, and these multi-types enhanced the cytotoxic activity of TRAIL [34].

One of the concerns for using MSCs as delivery vehicles is that the biological properties of MSCs may decreased [35]. Our data demonstrated that the release of soluble TRAIL did not change the biological properties of MSCs, which are consistent with previous reports that mesenchymal progenitors are resistant to TRAIL-mediated apoptosis [25,36].

TRAIL inhibits the proliferation of cancer cells, including acute myeloid leukemia cells [7,37]. We found that when both rhTRAIL and MSC secreted-TRAIL could inhibit the proliferation of B-ALL cells, MSC secreted-TRAIL showed a stronger inhibitory effect. The activation of PI3K/AKT pathway is important for proliferation of leukemia cells [9]; our data showed that MSC secreted-TRAIL suppressed PI3K/AKT pathway in B-ALL cells by downregulating the protein levels of leukemia proliferation-associated proteins PI3K, AKT, and the phosphorylation levels of MEK and ERK. Since TRAIL is to be used to inhibit tumor cells growth via apoptosis, we checked whether MSC secreted-TRAIL could induce apoptosis of B-ALL cells in vitro. Our data suggest that MSC secreted-TRAIL treatment induces the apoptosis of B-ALL cells, which is mainly mediated by the caspase-mediated cascade and mitochondria-mediated cascade (Figure 3F).

One of the disadvantages to using rhTRAIL for leukemia treatment is that cancer cells may be resistant to rhTRAIL. Some studies have reported that rhTRAIL may reduce the expression levels of TRAIL death receptors [28], which in turn makes tumor cells less sensitive to TRAIL [29]. We found that rhTRAIL downregulated the expression levels of TRAIL receptors TNFRSF10A and TNFRSF10B of B-ALL cells in vitro, whereas MSC secreted-sTRAIL did not reduce, but upregulated the expression levels of TNFRSF10A and TNFRSF10B. These data demonstrate that MSCs-secreted TRAIL may prolong the effects of TRAIL on B-ALL cells.

As we got encouraging data in vitro, additionally, we performed studies to investigate the effect of MSC secreted-sTRAIL on B-ALL in vivo. For this purpose, we used MSC-sTRAIL to treat B-ALL nude mice in vivo. Our data showed that the levels of B-ALL cells in both of rhTRAIL and MSC-sTRAIL treated mice were less than that in control PBS-treated mice, indicating that TRAIL could inhibit B-ALL cell proliferation in vivo.

Moreover, considering B-ALL cell could induce damages of major organs, it was possible to observe that TRAIL secreted by MSC may rescue the organ damage. Indeed, we found that the spleen and kidney of mice inoculated with B-ALL cells were enlarged compared with mice with no treatment. Further analyses showed that the spleen and kidney were damaged after B-ALL cell treatment, as the white pulp structures of the cells in the spleen were loosely arranged and were significantly damaged, and the texture of the medullary pay-off in the kidney was unclear. Interestingly, rhTRAIL or TRAIL secreted by MSCs could alleviate the B-ALL induced-spleen and kidney damage of SCID mice.

There are still more questions that need to be answered in future work. Firstly, whether TRAIL-induced B-ALL apoptosis is mediated by other pathways or by other biological changes of the cell needs to be further investigated. For example, previous studies have shown that gap junctions, mainly connexin proteins, were involved in carcinogenesis [38], and blocking gap junctions promoted the sensitivity of acute myeloid leukemia to chemodrugs [39]. Whether TRAIL could regulate the expression of gap junction proteins need to be investigated. Secondly, whether other kind of MSC, such as human amniotic fluid (hAF) MSCs, could be used as delivery system, and what is the difference between effects of hAF-MSC and of hUC-MSC need to be investigated. In pancreatic cancer, unmodified hAF-MSCs could inhibit the proliferation of cancer cells [40]; whether it can be used as delivery vehicle for TRAIL is still unknown. Thirdly, autologous MSCs, such as infrapatellar fat pad-derived MSC [41], has been shown to have better effects for disease treatment; as we used allogeneic MSC for our study, whether autologous MSCs showed similar effects as we reported here needs to be investigated.

## 4. Materials and Methods

### 4.1. Cell Culture

Nalm-6 cells (iCell Bioscience Inc, Shanghai, China) and Sup-b15 cells (Shenzhen Haodi Huatuo Biotechnology Co., Ltd., Shenzhen, China) were cultured as the ATCC reported. Human umbilical cord MSCs were purchased and expanded in maintenance medium (iCell Bioscience Inc., Cat#: HUM-iCell-e009). The differentiation of MSCs into adipocytes and osteogenesis have been described previously [42].

### 4.2. Animal

Male severe combined immune deficiency mice (SCID) mice (Guangzhou Huatuo Biotechnology Co., Ltd., Guangzhou, China) at 4–5 weeks of age were used. Mice at similar weights (18–19 g) were randomly grouped. Mice were housed in standard specific pathogen-free mouse rooms in Animal Service Center of Southwest Medical University with a 12-h dark-light cycle (7 a.m.–7 p.m.) with free access to water and regular chow diet. Mice were euthanized as reported previously [43]. Tissues were collected for preparation of histology analysis. All of the procedures were reviewed and approved by the Animal Welfare Committee of Southwest Medical University, and were performed in accordance with the animal care regulations of Southwest Medical University.

### 4.3. Cell Viability Assay

We investigated cell viability using CCK (Cell Counting Kit)-8 assay as manufacturer’s instructions. The absorbance at 450 nm was monitored using Microplate Reader (Bio-TeK, Winooski, VT, USA).

### 4.4. Lentivial Plasmid Construction and Transduction

Soluble TRAIL secreting lentiviral plasmid were generated and transduced into MSCs as described in Supplementary Materials.

### 4.5. Conditioned Medium Preparation

MSC-EV and MSC-conditioned medium were collected into 15-mL centrifuge tube and spun down (2000 rpm for 15 min), the supernatants were then collected and filtered through a 0.22 μm-diameter filter and syringe; then, the filtered supernatants were aliquoted into small volumes, and store at −80 °C for further assays or cell treatment.

### 4.6. Western Blotting

Total proteins were extracted and the protein concentration was measured as previous report [44]. A total of 30 μg of cell lysates was separated by SDS-PAGE gels and transferred to polyvinylidene difluoride (PVDF) membrane (EMD Millipore). For immunoblotting, the primary antibodies for GAPDH (1:2000; GENE TEX), PI3K (1:1000; SCBT), phosphorylated RAF-1 (1:1000; Millipore), phosphorylated MEK (1:1000; MILLIPORE), Bcl-2 (1:1000; ABZOOM), TRAIL (1:1000; Sino biological), P-ERK, P-AKT, PARP, Bax, Caspase 3, Caspase 8, or Caspase 9 from Cell Signaling Technologies, followed by anti-mouse lgG or anti-Rabbit lgG secondary antibodies (1:4000; Beyotime). The blots were analyzed by Developer (Gene Company Limited, Hongkong, China).

### 4.7. Measurement of TRAIL Levels by Enzyme-Linked Immunosorbent Assay (ELISA)

TRAIL levels of conditioned medium or mice serum were detected and quantified by TRAIL ELISA kit according to the manufacturer’s instructions and previous report [45].

### 4.8. Hematoxylin and Eosin (H&E) Staining

The tissues were immediately collected after the mice were sacrificed and fixed in 10% PBS-buffered formalin (pH 7.4) for 48 h. The following paraffin embedding, and H&E were performed by Sichuan Yuankangsheng Technology Co., Ltd. (Chengdu, China).

### 4.9. Data Analyses

GraphPad Prism 7.0 software (GraphPad Software) was used for statistical analyses. All data are represented as means ± SEM values of independent replicates. Student’s *t*-test was applied for statistical analyses. A *p* value of <0.05 was considered statistically significant.

## 5. Conclusions

In conclusion, our study demonstrated that MSC-sTRAIL could inhibit the proliferation and promote apoptosis of B-ALL cells in vitro and in vivo with no obvious side effects. Further studies may need to focus on investigating the appropriate MSC-sTRAIL doses used for the study. Meanwhile, to overcome the resistant to TRAIL, combinatory strategies with chemotherapy agents may be a good choice for B-ALL treatment. Overall, our study provided a potential strategy for B-ALL treatment in MSC delivered-TRAIL secretion. 

## Data Availability

Data is contained within the article and Supplementary Materials.

## Supplementary Materials

The following supporting information can be downloaded at: https://www.mdpi.com/article/10.3390/ph15111391/s1. Figure S1, rhTRAIL inhibits the proliferation of B-ALL cells, Nalm-6 and Sup-b15 cells; Figure S2, successful construction of MSC-sTRAIL lentiviral; Figure S3, secretion levels of sTRAIL of MSC-sTRAIL cells were increased in a time-dependent manner; Figure S4, MSC-sTRAIL does not downregulate TRAIL death receptor-associated mRNA expression; Figure S5, heart, liver, and lung weight/body weight ratio of all groups of mice.
