# Supplementary material for: Human Umbilical Cord MSC Delivered-Soluble TRAIL Inhibits the Proliferation and Promotes Apoptosis of B-ALL Cell In Vitro and In Vivo
Source: Pharmaceuticals (Basel). 2022 Nov 11;15(11):1391. doi: 10.3390/ph15111391 (PMC9693801; doi:10.3390/ph15111391)
Supplement: Supplementary file 1 [file pharmaceuticals-15-01391-s001.zip › pharmaceuticals-1937878-supplementary.pdf]

Supplementary data

Human Umbilical Cord MSC Delivered-Soluble TRAIL Inhibits the Proliferation and Promotes Apoptosis of B-ALL Cell In Vitro and In Vivo

Fangshan Chen [1,†], Xianmei Zhong [2,3,4,5,†], Qian Dai [2,3,4,†], Kuo Li [1], Wei Zhang [1], Jie Wang [1], Yueshui Zhao [2,3,4], Jing Shen [2,3,4], Zhangang Xiao [2,3,4], Hongyun Xing [6,*] and Jing Li [1,*]

1 Department of Oncology and Hematology, The Affiliated Traditional Chinese Medicine Hospital of Southwest Medical University, Luzhou 646000, China
2 Laboratory of Molecular Pharmacology, Department of Pharmacology, School of Pharmacy, Southwest Medical University, Luzhou 646000, China
3 Cell Therapy & Cell Drugs of Luzhou Key Laboratory, Luzhou 646000, China
4 South Sichuan Institute of Translational Medicine, Luzhou 646000, China
5 Department of Pharmacy, People's Hospital of Nanbu County, Nanchong 637300, China
6 Department of Hematology, The Affiliated Hospital of Southwest Medical University, Luzhou 646000, China
* Correspondence: xinghy52003@foxmail.com (H.X.); jing.li9@swmu.edu.cn (J.L.)
† These authors contributed equally to this work.

1. Materials and Methods

1.1 Chemicals and reagents

Roswell Park Memorial Institute (RPMI) Medium 1640 and Iscove's Modified Dulbecco Medium (IMDM) were purchased from Tianjin Haoyang biological products Technology Co., Ltd. Fetal bovine serum (FBS) was from Gibco. rhTRAIL was from Sino biological. The alkaline phosphatase (ALP) assay kits, oli red o staining kit are from Solarbio Life Sciences (China); the TRAIL enzyme linked immunosorbent assay (ELISA) assay Kit was from Multisciences (China). The primary antibodies were purchased from Cell Signaling Technologies (Danvers,MA), Sino biological, or MILLIPORE etc. The secondary antibodies were purchased from Beyotime (China).

1.2 Lentivial plasmid construction

The lentiviral expression vector PLV-3Flag-N-GreenPuro used in this experiment was provided by Clinical Medical Research Center, the Second Clinical Medical College of Jinan University, and the TRAIL plasmid was purchased from Hanheng Bio. We used the plasmid TRAIL as a template to amplify the gene fragment encoding TRAIL by PCR. (PCR procedure: 98℃ 3', 98℃ 5"-58℃ 15" - 72℃ 30" for 35 cycles, 72℃ 5', 12℃ ∞). The fragment was cloned into a lentiviral expression vector with a CMV promoter by enzymatic digestion and ligation, and the lentiviral expression vector was named PLV-3Flag-TRAIL-GreenPuro, and finally sequenced to determine whether the construction was successful. The plasmid TRAIL containing TRAIL sequence was used as a template for amplification using phusion high-Fidelity DNA Polymerase enzyme (Thero Scientific) with pTRAIL as the primer.

The pPLV-3Flag-TRAIL-GreenPuro plasmid was constructed (Supplementary data Figure 2). Based on the TRAIL plasmid, the sequence of interest TRAIL was flanked by restriction enzymes BamHI and NotI, and were amplified by PCR (Supplementary data Figure 2B). The plasmid pPLV-3Flag-

N-GreenPuro was used as the basis for double digestion by BamHI and NotI, and the double digestion product was recovered to obtain a linear vector of approximately 8237 bp in length. The target linear plasmid was then amplified using high-fidelity DNA polymerase and the linear fragment containing the BamHI and NotI digestion sites was recovered (Supplementary data Figure 2C). The above linear fragment was then double digested with BamHI and NotI. Next, the vector and the target fragment were ligated in the presence of T4 DNA ligase. The ligated products were transformed by E. coli DH5α, followed by plating and overnight incubation in 37℃ incubator to obtain the resistant clones, and the single clone was picked and expanded in LB liquid medium overnight, then, the plasmid was extracted for double digestionwith restriction endonucleases (BamHI and NotI) and DNA sequencing. As shown in supplementary data Figure 2D, of the two clonies picked, the correctly constructed plasmid could be cutted by BamHI and NotI. The lengths of the two fragments in the digested products were about 8237 bp and 860 bp, respectively, indicating that the lentiviral expression vector PLV-3Flag-TRAIL-GreenPuro was constructed successfully. The construction sequences were verified by commercial DNA sequencing.

1.3 MSC transduction

Properly sequenced sTRAIL gene has been then cloned into the lentiviral vector pPLV-3Flag-N-GreenPuro to gene modify MSCs. EV transduced MSCs were used as control. The 293T cells were transfected with pPLV-3Flag-TRAIL-N-GreenPuro using JetPEI DNA transfection reagent (PolyPlus-transfection, France) following manufacturer's instructions using a combination of lentiviral vector and helper plasmids: PsPAX2 and PMD2G. Lentiviral supernatant produced by transfected 293T supplemented with Protamine (SIGMA) was used to transduce UC-MSCs at early passages (P4). Viral supernatants were used either as fresh product or as -80℃ frozen product with. MSCs were

infected with lentivirus carrying empty vector or sTRAIL overeexpression-vector at different multiplicities of infection (MOI), the best MOI was determined by fluorescent assay. When MOI=20, positive GFP staining of hUCMSCs was estimated at >80% after 5 days of lentiviral infections (Figure 1A).

1.4 RNA isolation and real-time quantitative polymerase chain reaction (Q-PCR)

Total RNAs were extracted from Nalm-6 cells and Sup-b15 cells by Trizol (Invitrogen, CA) according to the manufacturer’s instructions. The quality and quantity of the RNA were determined by a 260/280 ratio using NanoDrop 2000 (Thermo Fisher Scientific). cDNAs were obtained by reverse transcribing 1 mg of total RNAs with FastKing RT Kit (Tiangen Biotech, China), which includes gDNase to remove potential genomic DNAs. Q-PCR were performed on a LightCycler® 480 instrument (Roche). The primer sequences for TNFRSF10A are as follows: forward: 5’-ACCTTCAAGTTTGTCGTCGTC-3’, reverse, 5’-CCAAAGGGCTATGTTCCCATT-3’; TNFRSF10B are as follows: forward: 5’-GCCCCACAAAAGAGGTC-3’, reverse, 5’-AGGTCATTCCAGTGAGTGCTA-3’; TNFRSF10C are as follows: forward: 5’-TCCCCAAGACCCTAAAGTTCG-3’, reverse, 5’-CAGTGGTGGCAGAGTAAGC-3’; TNFRSF10D are as follows: forward: 5’-TACCACGACCAGAGACACC-3’, reverse, 5’-CACCCTGTTCTACACGTCCG-3’; 18S are as follows: forward: 5’-AAGTCCCTGCCCTTTGTACACA-3’, reverse, 5’-GATCCGAGGGCCTCACTAAAC-3’. Results were normalized by the 18S mRNA and calculated using the $2^{-\triangle\triangle}$CT method as previous report [20].

1.5 Oil Red O staining

UC-MSCs were treated with adipogenic induction medium for 14 days. Following twice wash with PBS, the cells were fixed with 10% PBS-buffered formalin (pH 7.4) for 10 min. Oil Red O staining were then performed according to the manufacturer's instructions (Solarbio Life Sciences, China).

1.6 ALP staining

UC-MSCs were treated with osteogenic induction medium for 21 days. ALP levels in MSCs were detected by ALP kit according to the manufacturer's instructions (Solarbio Life Sciences, China).

1.7 Wright-Giemsa staining

Bone marrow smears were taken from the femur of each group of mice. After the smears were dried, Wright-Giemsa staining solution (Beijing Wokai Biotechnology Co., Ltd) was added dropwise. The smears were stained for 3-10 min, following rinse under running water and left to dry. The dried smears were checked under a microscope.

2. Results

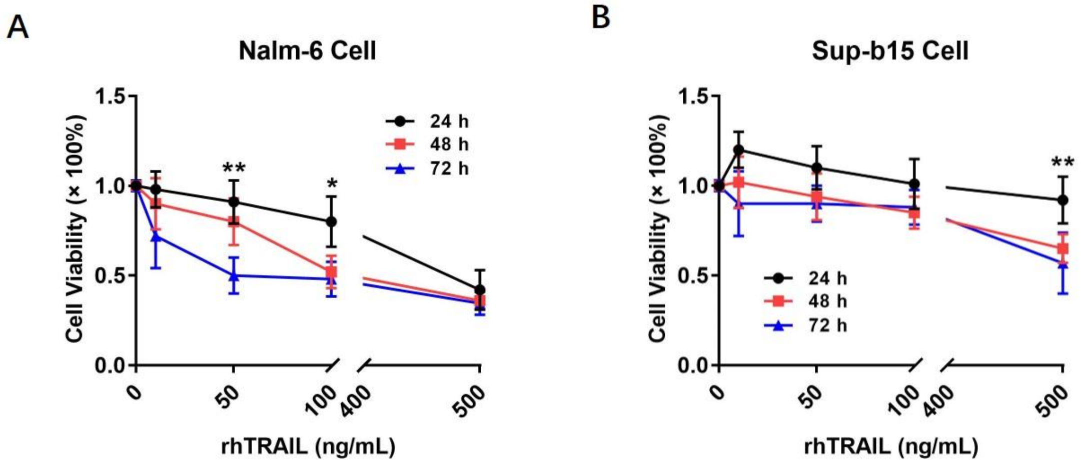

Supplementary Figure S1. rhTRAIL inhibits the proliferation of B-ALL cells, Nalm-6 and Sup-b15 cells. After treating Nalm-6 and Sup-b15 cells with 0 ng/ml, 10 ng/ml, 50 ng/ml, 100 ng/ml, 500 ng/ml rhTRAIL for 24 h, 48 h, or 72 h, cell viability were detected by CCK-8 assay. (*n*=6 per group, Student's t test, *, P<0.05, **, P<0.01, 24 h group compared with 72 h group)

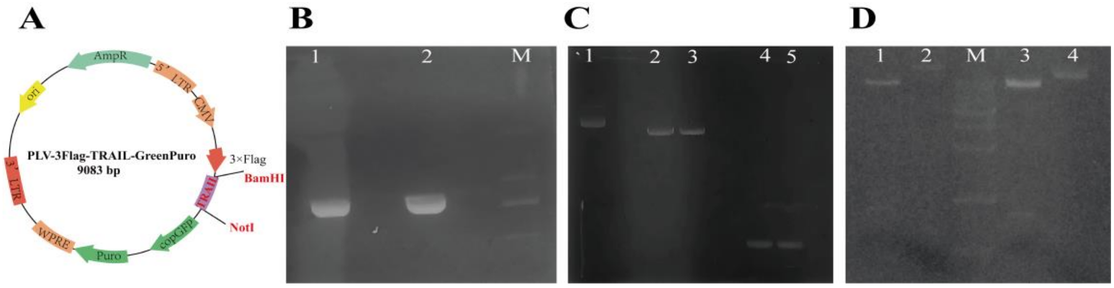

Supplementary Figure S2. Successful construction of MSC-sTRAIL lentiviral vector. **(A)** Constructed pPLV-3Flag-TRAIL-GreenPuro plasmid. **(B)** TRAIL plasmid PCR amplification product. **(C)** BamHI and NotI double enzymolysis plasmids pPLV-3Flag-N-GreenPuro and the corresponding products of TRAIL (1 before the enzymolysis of pPLV-3Flag-N-GreenPuro vector, 2 and 3 after the enzymolysis of pPLV-3Flag-N-GreenPuro vector, and 4 and 5 after the enzymolysis of the target fragment of TRAIL). **(D)** The transformation results of the ligation product pPLV-3Flag-TRAIL-GreenPuro were identified by bacterial plasmid PCR (2 and 4 represent different clones selected, and 1 and 3 represent double enzyme digestion of different clones selected. M stands for DNA marker.)

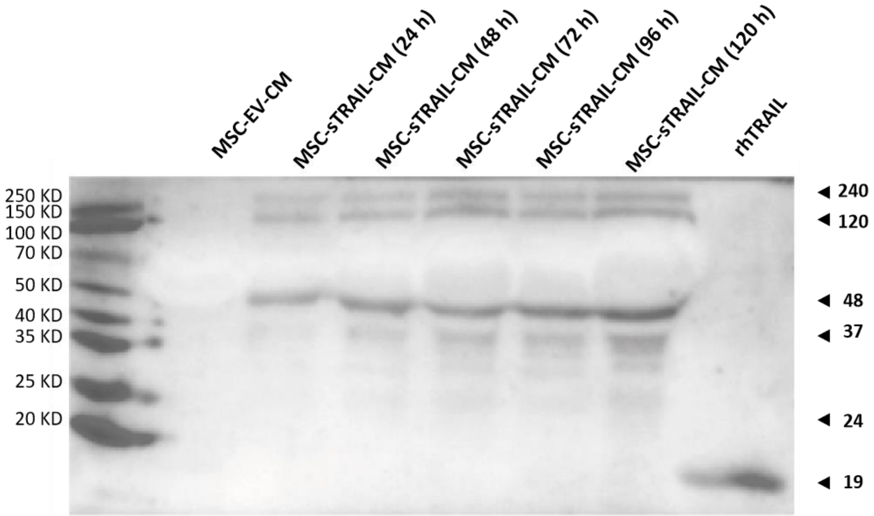

Supplementary Figure S3. Secretion levels of sTRAIL of MSC-sTRAIL cells were increased in a time-dependent manner. The MSC-EV and MSC-sTRAIL cells were placed on 100-mm cell culture dishes (5 X 10^5 cells/dish), then, we collected conditioned medium after 24 h, 48 h, 72 h, 96 h, and 120 h of plating, respectively, then, the levels of TRAIL were checked by western blotting.

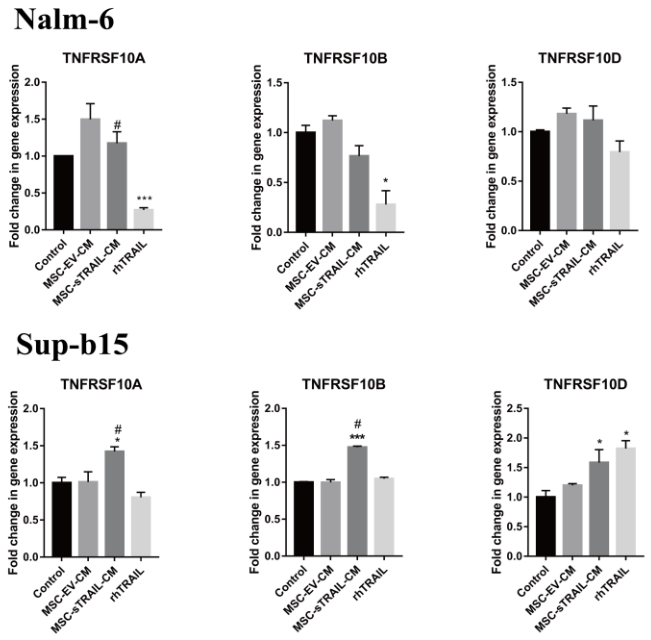

Supplementary Figure S4. MSC-sTRAIL does not downregulate TRAIL death receptor-associated mRNA expression. Compared with control, rhTRAIL decreased the expression of TRAIL death receptor (TNFRSF10A and TNFRSF10B) mRNA in Nalm-6 cells. MSC-sTRAIL does not decrease TRAIL death receptor (TNFRSF10A and TNFRSF10B) mRNA expression in Nalm-6 cells. MSC-sTRAIL increased TNFRSF10A mRNA expression in Nalm-6 cells compared to rhTRAIL. There was no significant change in TNFRSF10D mRNA expression in Nalm-6 cells in all groups. MSC-sTRAIL and rhTRAIL upregulated the expression of TNFRSF10D mRNA in Sup-b15 cells compared with control. MSC-sTRAIL increased the expression of TRAIL death receptor (TNFRSF10A and TNFRSF10B) mRNA in Sup-b15 cells compared to rhTRAIL. (n=3 per group; Student's t test, *, *P<0.05*, **, *P<0.01*, ***, *P<0.001*, MSC-EV-CM group, MSC-sTRAIL-CM group and rhTRAIL compared with control group; #, *P<0.05*, ##, *P<0.01*, ###, *P<0.001*, MSC-sTRAIL-CM group compared with rhTRAIL group)

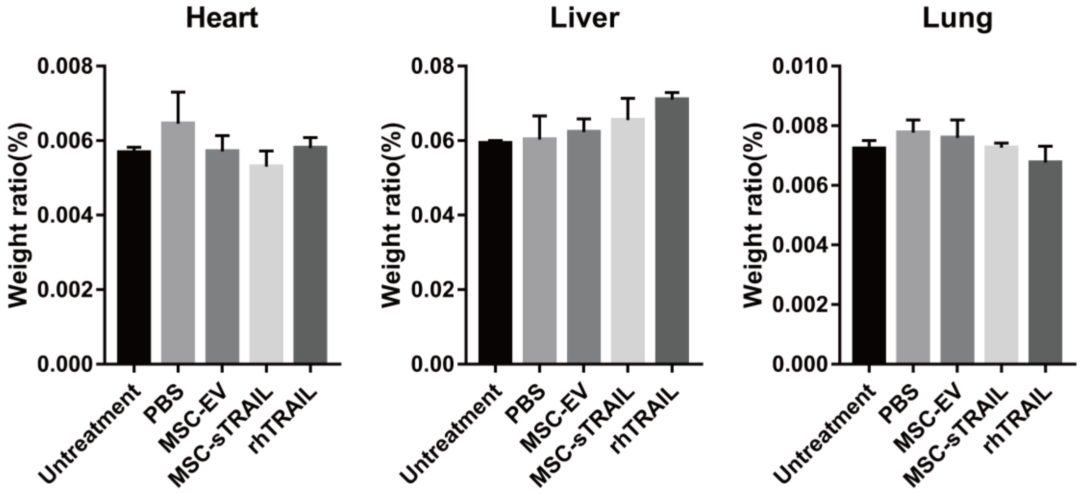

Supplementary Figure S5. Heart, liver, and lung weight/body weight ratio of all groups of mice. No significant changes in heart, liver and lung weight/body weight ratios were observed in all groups of mice.

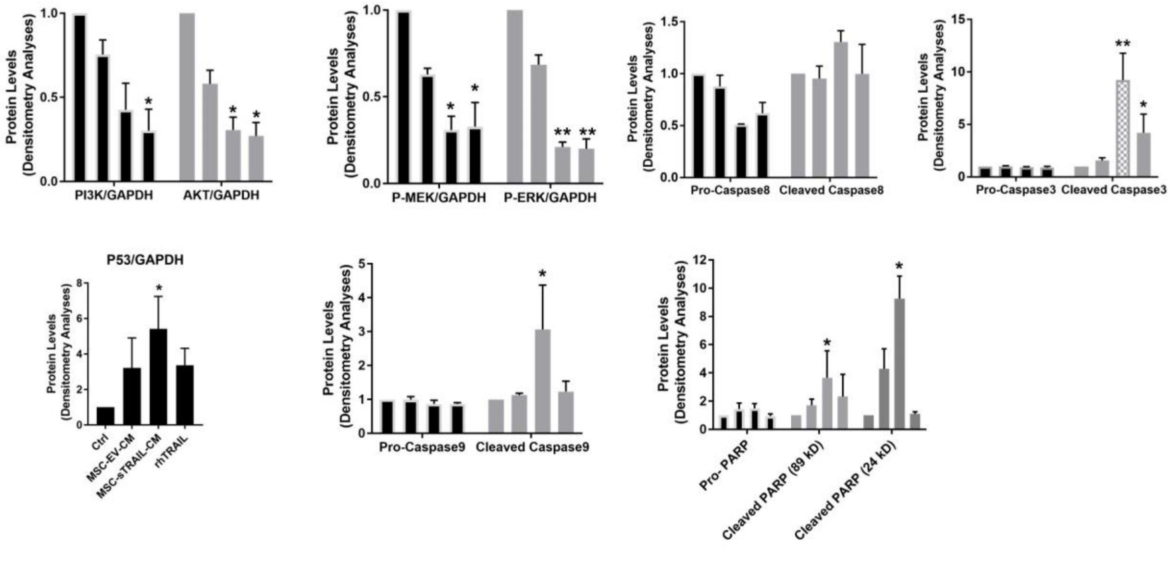

Supplementary Figure S6. Densitometry analyses of the western blotting results of figure 2 & 3. GAPDH was used as inner control; the densitometry readings of each band were calculated by Image J software, then the ratio of target protein/GAPDH were calculated and compared between each group. (*n*=3 independent experiments per group; Student's *t* test, *, *P*<0.05, **, *P*<0.01, MSC-sTRAIL-CM group, and rhTRAIL group compared with MSC-EV-CM group).
